# Factors associated with men’s health facility attendance as clients and caregivers in Malawi: a community-representative survey

**DOI:** 10.1186/s12889-022-14300-8

**Published:** 2022-10-12

**Authors:** Marguerite Thorp, Kelvin T. Balakasi, Misheck Mphande, Isabella Robson, Shaukat Khan, Christian Stillson, Naoko Doi, Brooke E. Nichols, Kathryn Dovel

**Affiliations:** 1 Division of Infectious Diseases David Geffen School of Medicine, University of California – Los Angeles, 10833 Le Conte Blvd CHS 37-121, 90095 Los Angeles, CA USA; 2 Partners in Hope, Lilongwe, Malawi; 3 Clinton Health Access Initiative, Boston, USA; 4 Boston University School of Public Health, Boston, USA; 5 Division of Infectious Diseases, University of California – Los Angeles, Los Angeles, USA

## Abstract

**Introduction:**

Men have higher rates of morbidity and mortality across nearly all top ten causes of mortality worldwide. Much of this disparity is attributed to men’s lower utilization of routine health services; however, little is known about men’s general healthcare utilization in sub-Saharan Africa.

**Methods:**

We analyze the responses of 1,116 men in a community-representative survey of men drawn from a multi-staged sample of residents of 36 villages in Malawi to identify factors associated with men’s facility attendance in the last 12 months, either for men’s own health (client visit) or to support the health care of someone else (caregiver visit). We conducted single-variable tests of association and multivariable logistic regression with random effects to account for clustering at the village level.

**Results:**

Median age of participants was 34, 74% were married, and 82% attended a health facility in the last year (63% as client, 47% as caregiver). Neither gender norm beliefs nor socioeconomic factors were independently associated with attending a client visit. Only problems with quality of health services (adjusted odds ratio [aOR] 0.294, 95% confidence interval [CI] 0.10—0.823) and good health (aOR 0.668, 95% CI 0.462–0.967) were independently associated with client visit attendance. Stronger beliefs in gender norms were associated with caregiver visits (beliefs about acceptability of violence [aOR = 0.661, 95% CI 0.488–0.896], male sexual dominance [aOR = 0.703, 95% CI 0.505–0.978], and traditional women’s roles [aOR = 0.718, 95% CI 0.533–0.966]). Older age (aOR 0.542, 95% CI 0.401–0.731) and being married (aOR 2.380, 95% CI 1.196–4.737) were also independently associated with caregiver visits.

**Conclusion:**

Quality of services offered at local health facilities and men’s health status were the only variables associated with client facility visits among men, while harmful gender norms, not being married, and being younger were negatively associated with caregiver visits.

**Supplementary Information:**

The online version contains supplementary material available at 10.1186/s12889-022-14300-8.

## Introduction

Men experience disproportionately high rates of morbidity and mortality compared to women across nearly all top ten causes of disease worldwide. [1] In southern and eastern Africa, gender disparities in HIV and tuberculosis (TB) outcomes are particularly stark – in 2016, men accounted for only 40% of people living with HIV but represented 54% of those who died of AIDS. [2].

Regular engagement with health systems can improve poor health outcomes for men. Routine facility visits may increase men’s comfort level with health systems [3] and can provide critical entry points for men to access screening services (such as for HIV, TB, or various non communicable diseases [NCDs]), preventative care, or early-stage care for illness. [4] While men in sub-Saharan Africa are not generally encouraged nor expected to attend health facilities except for HIV testing, [5] a growing number of studies show that men do attend facilities frequently, although their attendance is less visible than women’s. [6, 7] A recent study from Malawi showed that over 80% of men visited a health facility in the past 12 months, most attending outpatient departments for acute needs. Interestingly, the majority had attended facilities as both clients and caregivers during this time period. Over 45% of men attended a health facility to support friends’ or family members’ use of health services (caregiver visits). [6] Such facility visits could provide key entry points for key non-acute services, although such integrated care is poorly implemented to date. [8, 9].

While the majority of men appear to attend facilities for acute care, it is unclear if certain sub-populations of men do *not* attend general facility visits and what factors are associated with men’s general facility attendance (either for their own health or as caregivers). This question is important both for ensuring equity in men’s health and determining if men’s routine facility visits can be used to as an entry point for other priority services. For example, if facility services systematically reach all men, facility visits could be optimized as a primary entry point for improving population-level coverage for HIV, TB, and NCD screening among men. However, if facility services systematically exclude sub-populations of men, outreach services will likely be required to achieve population-level coverage. Both client and caregiver visits are potential entry points for additional services. [10, 11] Throughout the region, caregiver visits have been a critical entry point for women’s health education and screening services. [12, 13] The same could be done for men if a large portion of men attend facilities as caregivers. [14].

Research from HIV and TB services examines factors associated with service utilization and offers a useful system for categorizing potential factors that might also influence men’s general facility attendance. [15, 16] *Demographic* characteristics, such as education, age, marital status, income, and dependence on day labor, are all associated with use of HIV testing. [7, 17–22] *Harmful gender norms* regarding masculinity are also found to negatively influence men’s use of HIV and TB services, although most of the literature relies on qualitative data. [23, 24] Finally, *health system* factors such as quality of services, length of time required to receive services, and days/times when services are offered are associated with men’s use of reproductive health services. [25, 26] There is evidence that these same factors may dissuade men from attending as caregivers. [27 ] However, the above factors might not be associated with men’s general facility attendance. Most men attend facilities for curative care for non-stigmatized illnesses [6] – the acute and non-stigmatized nature of illness for most curative services may mitigate barriers traditionally experienced for HIV and TB services.

We assessed individual- and facility-level factors associated with men’s attendance to a health facility in the past 12 months, using data from a cross-sectional, community representative survey with men in rural Malawi. We examined factors associated with client visits (seeking care for men’s own health) and caregiver visits (providing support for someone else’s health).

## Methods

### Setting

Malawi is a predominantly rural country in southern Africa with an HIV prevalence of 13.2% in the Southern region and 5.7% in the Central region. [28] Basic primary health services, including sexual and reproductive health care, HIV services, and TB care, are free at all Ministry of Health and mission facilities. Acute care and other outpatient services are free at Ministry of Health facilities, but at mission facilities are offered at cost. Health insurance plays a negligible role in health access in Malawi; it comprises less than 5% of total health expenditures and, without a national health insurance scheme, typically only formally employed Malawians have insurance. [29].

### Design

We use data from a large cross-sectional, community representative survey with men in central and southern Malawi collected from 15 August to 18 October 2019. The parent study examined the frequency with which men attend health facilities (for any reason) and coverage of HIV testing services at these visits. Detailed information of the parent study has been published elsewhere. [6] Briefly, the study used a multi-staged sampling design. First, we purposively selected two of Malawi’s most populous districts in the central and southern regions and three mid-size health facilities per district. Second, we randomly selected 6 villages within each facility catchment area (36 villages in total) and roughly 45 male respondents per village. Household census listings from each village were used to randomly select respondents using randomized number generation. Random selection within each village was stratified by age categories: young men (15-24-years, n = 300); middle-aged men (25-39-years, n = 425); and older men (40+-years, n = 425).

Eligibility criteria for individual men were: (1) aged 15–64 years; (2) current resident of the participating village; and (3) spent > 15 nights within the village in the past 30 days. Exclusion criteria included: (1) men who did not meet eligibility criteria, (2) men who were drunk, disabled, or otherwise unable to consent, and (3) men who did not match randomization identifiers. For this secondary analysis, we also exclude men who self-report as ever testing HIV-positive, because their health service utilization would not represent the general population and we would anticipate increased facility visits for HIV treatment services.

### Data collection

Surveys were conducted with all randomly selected men, with the assistance of community health workers and village chiefs for identification. Survey domains included: (1) recent facility visits, including quality-related experience during the visit like wait time and privacy; (2) sociodemographic characteristics and health status; (3) gender norms; and (4) HIV testing history. The survey tool was developed in English and translated into the local language (Chichewa). It was piloted with approximately 25 men who met eligibility criteria and modified as needed for clarity. Surveys lasted approximately 55 min on average.

### Variables

For this secondary analysis, our primary outcome of interest was facility visit in the past 12 months. Participants were asked to describe their four most recent visits to a health facility, including who received the primary health service at that visit. We created a dichotomous variable for having at least one facility visit (not for HIV treatment) within the past 12 months, distinguishing between client visits and caregiver visits.

We drew from HIV and TB literature to identify potential factors associated with men’s general facility attendance to include in the model. [17–21] Sociodemographic characteristics included ever attending secondary school (yes/no), currently having children living at home (yes/no), having financial savings at the time of the survey (yes/no), currently employed (yes/no), mobility (yes/no), and a household wealth index scale. We defined employment as either formally employed or self-employed over the past 12 months, while unemployment included both unemployment and *ganyu* work, a form of daily wage labor without long-term predictability. Mobility was defined as spending more than 3 nights away from home in the past 6 months. For the household wealth index, we used the first dimension of a principal component analysis of 22 household assets including items such as a chair, a radio, and a bicycle. [30] To make the index more easily interpretable, we linearly transformed it to a scale of 0 to 10, with a resulting mean of 1.88.

Men’s acceptance of harmful gender norms has been identified as a barrier to HIV and TB services in qualitatively studies. [24–26] To measure men’s acceptance of harmful gender norms, we use 12 questions from the Gender Equitable Men (GEM) survey, a validated tool used widely throughout sub-Saharan Africa. [31–33] While the tool has not been fully validated in Malawi, it has been validated in the region and has been used in other studies in Malawi. [32, 34] Questions were asked on a 5-point Likert scale from “strongly agree” to “strongly disagree.” We collapsed questions responses into 4 distinct measures, with 3 questions in each measure: *measure 1*: violence is permissible; *measure 2*: male sexual dominance is acceptable; *measure 3*: women’s roles should be confined to the household; and *measure 4*: men control household decisions, which was not scored on a Likert scale, with participants receiving scores of 1 for “male only,” 2 for “joint decision,” and 3 for “female only” on questions regarding who made decisions within respondents’ own household (see Appendix A for specific questions). We summed participant scores for each question in the construct (based on the Likert scale). We then created a dichotomous variable to measure respondents’ relative acceptance of harmful gender norms as compared to other study participants, separating the 20% of respondents with the highest degree of gender bias from the remaining 80% in each category. We found no concerning evidence of multicollinearity between the four gender norm constructs using variance inflation factors (all VIF < 2.0).

Quality of health services is associated with service utilization across numerous disease categories and conditions. [35–37] We included a composite measure for quality of services offered at respondents’ closest public facility. Participants were asked about their satisfaction with services received, using questions from the Service Provision Assessment (SPA) [38] that covered service availability (wait time and opening hours), privacy (ability to discuss concerns and privacy of their discussion and of the examination), medicine availability, and cleanliness. Participants were asked about whether they experienced problems during the visit in each domain (see Appendix B for all satisfaction questions). There were six major health facilities within the survey catchment area, with an average of 115 respondents reporting on the quality of health services at each facility (range 61–163 respondents). We generated a composite quality score (maximum of 7 problems) by averaging the problems reported within each domain for all men who described a visit to that facility. Facility scores were then applied to each respondent living in the catchment area of that respective facility, regardless of whether they reported visiting that facility.

### Analysis

We used Wilcoxon rank sum tests, t-tests, and Chi-square tests to examine factors associated with facility attendance. Factors that had a p-value of < 0.10 in univariable analysis were included in our multivariable model. For client visits, we also included two control variables, age and self-reported health status, regardless of their association in the single-variable analyses, because we believe those to be intrinsically related to the need for a clinic visit. The multivariable model was a logistic regression with random effects to account for clustering at the village level, and we report results at the p < 0.05 significance level. Clustering at facility level (n = 6) did not notably changes results. Analyses were completed in Stata v.14. [39].

### Human subjects

The parent study was approved by the National Health Sciences Review Committee (NHSRC) of Malawi and the University of California Los Angeles (UCLA) Institutional Review Board. Written, informed consent was ascertained from all respondents; written, informed assent was attained from respondents and written, informed consent was obtained from parents or legal guardians for participants between 15 and 17 years.

## Results

Our analysis included 1,116 respondents after excluding men who reported being HIV-positive. Over 74% (824/1116) of participants were married, 88% (983/1116) owned land (not shown), and 20% (228/1116) attended at least some secondary school. A total of 82% (919/1116) of participants attended at least one facility visit in the past 12 months: 63% (701/1116) had at least one client visit, while 47% (524/1116) attended at least one visit as a caregiver in the past year (see Table 1). Interestingly, 25% of participants attended a health facility besides their local Ministry of Health facility, meaning they either had to travel a longer distance or had to pay user fees for a private facility (analysis not shown). The mean distance from facilities to village was 5.11 km with a standard deviation of 3.46 km.

There were few significant differences between participants who attended client visits in the last year and those who did not. Within sociodemographic and gender norms variables, only household assets trended toward significance (mean household asset score of 1.95 among those who attended a client visit versus 1·76 among those who did not; p = 0.09). The participant’s distance from facility was associated with the likelihood of a client visit in single-variable analysis (p < 0.001).

Perceived quality of services offered at local health facilities was significantly associated with attending a client visit in the past 12 months. Men who lived in catchment areas of health facilities with more frequently reported quality problems were less likely to attend client visits (problem score of 1.19 among those who attended a visit versus 1.26 among those who did not, out of a maximum of 7; p < 0.001).

Factors associated with facility attendance differed for caregiver visits. Scoring in the top quintile of respondents on each of the four beliefs regarding harmful gender norms was negatively associated with men’s attendance to caregiver visits: participants who believed men should assert violence to get their way (19% of men who attended a caregiver visit were in the top 20th percentile on the violence measure versus 29% among those who did not attend caregiver visits; p = 0.002), participants who believed men have natural sexual dominance (17% versus 24%; p = 0.006), and participants who believed household or childcare duties were strictly women’s roles (24% versus 32%; p = 0.004) were all less likely to attend caregiver visits. The male participant’s control over household financial decisions was associated with a higher likelihood of attending a caregiver visit (30% versus 25%; p = 0.054). Being formally employed or self-employed, versus being unemployed or relying on piece work, was associated with men attending a caregiver visit in the past 12 months (65% versus 55%; p = 0.001). Unlike client visits, attending caregiver visits was not associated with local facility quality of care metrics.

**Table 1:** Single-variable analysis of factors associated with clinic visits in previous 12 months *A table comparing characteristics of men in the whole study sample, men who had and had not attended a client visit in the last 12 months, and men who had and had not attended a caregiver visit in the last 12 months.*

Variable			Client visits in last 12 months					Caregiver visits in last 12 months				
	**Overall**		**Attended client visit**		**No client visit**		**P value**	**Attended caregiver visit**		**No caregiver visit**		**P-value**
	**n**	**(%)**	**n**	**(%)**	**n**	**(%)**		**n**	**(%)**	**n**	**(%)**	
All men	1116	(100%)	701	(63%)	415	(37%)		524	(47%)	592	(53%)	
**Harmful Gender Norm Beliefs**												
Violence scale - Top 20%	272	(24%)	168	(24%)	104	(25%)	0.68	101	(19%)	171	(29%)	0.00
Dominance scale - Top 20%	231	(21%)	135	(19%)	96	(23%)	0.12	90	(17%)	141	(24%)	0.01
Women’s roles scale - Top 20%	315	(28%)	205	(29%)	110	(27%)	0.33	126	(24%)	189	(32%)	0.00
Decision-making scale - Top 20%	308	(28%)	203	(29%)	105	(25%)	0.19	159	(30%)	149	(25%)	0.05
**Sociodemographic Indicators**												
*Age* ‡							0.60					0.00
15–29 years	345	(31%)	224	(32%)	121	(29%)		126	(24%)	219	(37%)	
30–49 years	393	(35%)	245	(35%)	148	(36%)		230	(44%)	163	(28%)	
50 + years	378	(34%)	232	(33%)	146	(35%)		168	(32%)	210	(35%)	
*Household composition*												
Married	824	(74%)	511	(73%)	313	(75%)	0.35	440	(84%)	384	(65%)	0.00
Has children at home	820	(73%)	508	(72%)	312	(75%)	0.32	437	(83%)	383	(65%)	0.00
*Distance from facility (km)*	5.11		4.71		5.80		0.00	5.11		5.12		0.41
*Economic indicators*												
Assets index - mean score	1.88		1.95		1.76		0.09	1.86		1.90		0.43
Formal or self-employment	663	(59%)	417	(59%)	246	(59%)	0.95	338	(65%)	325	(55%)	0.00
Mobility (> 3 nights away/6 mo.)	299	(27%)	197	(28%)	102	(25%)	0.20	151	(29%)	148	(25%)	0.15
Has savings	356	(32%)	230	(33%)	126	(30%)	0.40	178	(34%)	178	(30%)	0.16
Attended secondary school	228	(20%)	152	(22%)	76	(18%)	0.18	110	(21%)	118	(20%)	0.66
*Health Status*												
Good or very good health	941	(84%)	583	(83%)	358	(86%)	0.17	447	(85%)	494	(83%)	0.39
**Health system Factors**												
Quality problems composite (maximum 7)	1.22		1.19		1.26		0.00	1.22		1.22		0.72
Service availability problems (2)	0.58		0.56		0.60		0.00	0.58		0.57		0.16
Privacy problems (3)	0.24		0.24		0.25		0.09	0.24		0.24		0.26
Medicine availability prob. (1)	0.27		0.27		0.28		0.07	0.27		0.27		0.74
Cleanliness problems (1)	0.12		0.12		0.13		0.00	0.12		0.12		0.76

‡ Included in multivariable model as a control regardless of significance in single-variable analysis

In our multivariable model for client visits (see Table 2), none of the wealth or demographic characteristics were associated with client visits, including distance from facility. Quality of health services offered at local facilities was significantly associated with visits: problems with overall quality was negatively associated with men’s likelihood of attending a client visit in the past 12 months (adjusted odds ratio [aOR] 0.294, 95% confidence interval [CI] 0.105–0.823) when controlling for wealth, demographics, self-rated health, and mixed effects from village level clustering. Self-rated good health was also negatively associated with client visits (aOR 0.668, 95% CI 0.462–0.967).

For caregiver visits, men with the most strongly-held harmful beliefs regarding three of the four gender norm measures remained significantly less likely to attend a caregiver visit (violence [aOR = 0.661, 95% CI 0.488–0.896], sexual dominance [aOR = 0.703, 95% CI 0.505–0.978], and women’s roles [aOR = 0.718, 95% CI 0.533–0.966]). Tests for collinearity showed no evidence that the gender norms were highly collinear. Age and marital status were also significantly associated with caregiver visits, with men over age 50 less likely to make caregiver visits than younger men (aOR 0.542, 95% CI 0.401–0.731) and married men more likely to make caregiver visits than non-married men (aOR 2.380, 95% CI 1.196–4.737). Employment status was not significant in the multivariable model.

The same analysis was conducted to understand factors associated with any visit (either client or caregiver) and no new independent factors were observed in the model (see Appendix C).

**Table 2:** Multivariable model of factors associated with clinic visits in previous 12 months *Two multivariable analyses: one model shows factors associated with the likelihood of a man having a client visit in the last 12 months, and a second model shows factors that are associated with the likelihood of a caregiver visit in the last 12 months*

Variable	Likelihood of CLIENT visit		Likelihood of CAREGIVER visit		
	**Odds ratio**	**95% CI**	**Odds ratio**	**95% CI**	
**Harmful Gender Norm Beliefs**					
Violence scale			0.661 ***	0.488–0.896	
Dominance scale			0.703 **	0.505–0.978	
Women’s roles scale			0.718 **	0.533–0.966	
Decision-making scale			0.944	0.702–1.267	
**Sociodemographic Indicators**					
*Age (vs. age 30–49) ‡*					
Young adult (15–29 years)	1.154	0.838–1.590	1.093	0.679–1.759	
Older adult (50 + years)	0.938	0.688–1.279	0.542 ***	0.401–0.731	
*Household composition*					
Married			2.380 **	1.196–4.737	
Has any children living at home			1.812	0.877–3.746	
*Distance from nearest public facility*	0.980	0.924–1.039			
*Economic Indicators*					
Wealth score	1.039	0.951–1.133			
Employment (formal or self-employed vs. unemployed or ganyu)			1.113	0.828–1.497	
Good or very good health (self-reported health) ‡	0.668 **	0.462–0.967			
**Health system Factors**					
Problems with overall quality (composite)	0.294 **	0.105–0.823			
** Significant at 0.05					
*** Significant at 0.01					
‡ Included in model as a control regardless of significance in single-variable analysis					

## Discussion

We used data from a community-representative, cross-sectional survey with men in Malawi to understand factors associated with men’s attendance to health facilities within the past 12 months. Understanding which men are missed by general facility visits is critical to understand the role of integrated services for bridging the gap in men’s health care. We find that for client visits (whereby men access services for their own health), poor quality health services at local health facilities and feeling healthy at the time of the survey were negatively associated with facility visits; sociodemographic factors and harmful gender norms were not associated with client visits. For caregiver visits (whereby men support the health care of others), ascribing to harmful gender norms, being ≥ 50 years of age, and being unmarried were negatively associated with facility visits. Findings suggest that men’s general facility attendance as clients is, on the whole, equitable across a broad range of rural Malawian men. Men’s client visits could provide an equitable venue for increasing access to key services (such as HIV and TB screening) among men at the population level, without missing key sub-populations.

The lack of association between client visits and demographic and individual-level characteristics (such as age, economic status, or gender norms) is in contrast to the HIV and TB literature that shows poverty, low educational attainment, and harmful beliefs about gender norms are all negatively associated with men’s use of high-priority services. [18, 21, 24] Previous findings from Malawi using the same dataset found that 83% of men’s client visits are to outpatient departments for acute or curative care services, [6] suggesting that curative care may not have the same barriers as HIV and TB screening services. Divergent findings between men’s general facility attendance and HIV / TB services may also be impacted by how health services and HIV services are often organized around women’s and children’s health, which can create additional barriers to care that may not be present within outpatient departments. [3].

We found that poor quality health services at local health facilities was negatively associated with men’s general facility attendance for client visits. This finding highlights the importance of the clinic experience for continued use of care. Other research shows how quality is associated with utilization of services such as facility deliveries, [36] primary care, [35] and HIV care. [37] Future studies should include metrics on service quality when studying men’s use of health services in order to further understand this relationship.

This is one of the few studies to our knowledge to assess factors associated with men’s caregiver visits, outside of studies exclusively focused on prevention of mother-to-child transmission. Interestingly, we found very different factors associated with client versus caregiver visits. Client visits are motivated by men’s own health, often by an acute illness or injury. [6] Most men, regardless of demographics or beliefs about gender norms, may choose to seek curative care to receive immediate relief. Caregiver visits, however, are for the well-being of others, [40, 41] and men are rarely the only available caregivers – other family members may be able to perform the role of a caregiver so men can continue other activities. It is therefore unsurprising that the men who do choose to serve as caregivers have less restrictive views of gender norms and may value caregiving as a reasonable priority over income generation. [42, 43] Though our results show that caregiver visits are not made equally by all men, the result that 47% of participants had made a caregiver visit in the last year suggests these events may provide opportunities to familiarize men with the health care system and offer screening services.

Our findings challenge the notion that harmful beliefs regarding gender norms universally discourage men’s use of health services. [23, 24] The fact that gender norms were *not* associated with client visits, but were associated with caregiver visits, suggests that gender norms do not constrain all health-seeking behavior. Our results highlight that masculinity is one component among many in men’s dynamic decision-making process regarding engagement with health facilities. [26].

Our study has several limitations. First, our data relies on self-report and may be sensitive to social desirability and recall bias. Social desirability bias could affect men’s report of gender norm beliefs and health-seeking behavior, reducing our ability to detect relationships between gender norms and health-seeking behavior. However, because there was a clear and strong association between gender norm beliefs and caregiver visits, we are more confident in our null result for client visits. Recall bias may affect men’s recollection of the quality of services, though the effect should be minor for activities in the last 12 months and should affect all groups of men similarly. Second, and perhaps most importantly given our conclusions, our quality-of-care metrics are based on reports of respondents in this study. We considered alternative data sources, such as the Demographic Health Surveys Program’s Service Provision Assessment (SPA) data, but we felt that community *perceptions* of quality would be at least as relevant (if not more so) than official measures. In total, our respondents described an average of 115 visits per facility (range 61–163), many more than the SPA is able to observe. Third, our sampling frame was not designed for varying village size or population age distribution. The parent study conducted a sensitivity analysis using weights for village size and found no difference. [6] Finally, we did not ask about presence of pregnant people or older adult dependents living in respondents’ household, which may be positively associated with men making caregiver visits.

## Conclusion

Factors associated with men’s facility attendance are nuanced and vary by the type of visit made – men’s facility attendance for their own health was only associated with quality of services available to them (and by their self-reported health), whereas men’s attendance as caregivers was associated with men’s strong acceptance of harmful gender norms. These findings suggest that client visits could be an entry point to reach the general male population. Our analysis also suggests that health system improvements may be the best tool to engage men in general health care.

## Electronic supplementary material

Below is the link to the electronic supplementary material.

Supplementary Material 1

Supplementary Material 2

Supplementary Material 3

## Data Availability

Data is not currently available in a repository due to the sensitive information included in survey responses. Data is available to individuals by request via email to the corresponding author.
