# Supplementary material for: Factors associated with men’s health facility attendance as clients and caregivers in Malawi: a community-representative survey
Source: BMC Public Health. 2022 Oct 12;22:1904. doi: 10.1186/s12889-022-14300-8 (PMC9558411; doi:10.1186/s12889-022-14300-8)
Supplement: Supplementary file 1 — Supplementary Material 1 [file 12889_2022_14300_MOESM1_ESM.pdf]

## Appendix: Gender Norm Survey Questions

| | |
|---|---|
| *Permissibility of Violence*<br>There are times when a woman deserves to be beaten.<br>A woman should tolerate violence in order to keep her family together.<br>If someone insults me, I will defend my reputation, with force if I have to.<br><br>*Men's Sexual Dominance*<br>Men need sex more than women.<br>Men don’t talk about sex, they just do it.<br>Men are always ready to have sex.<br><br>*Women's Roles*<br>Changing diapers, giving kids a bath & feeding kids are mother’s responsibility.<br>It is a woman’s responsibility to avoid getting pregnant.<br>A man should have the final word about decisions in his home. | Likert scoring:<br><br>1 - Strongly agree<br>2 - Agree<br>3 - Neutral<br>4 - Disagree<br>5 - Strongly disagree |
| *Household Decision-Making*<br>Who usually decides how the money you earn will be used?<br>Who usually decides how your partner's earnings will be used?<br>Who usually makes decisions about making major household purchases? | Scoring:<br>1 - Myself<br>2 - Jointly (partner & self)<br>3 - Partner (or other) |
