# Supplementary material for: Factors associated with men’s health facility attendance as clients and caregivers in Malawi: a community-representative survey
Source: BMC Public Health. 2022 Oct 12;22:1904. doi: 10.1186/s12889-022-14300-8 (PMC9558411; doi:10.1186/s12889-022-14300-8)
Supplement: Supplementary file 2 — Supplementary Material 2 [file 12889_2022_14300_MOESM2_ESM.pdf]

Appendix: Quality Problem Survey Questions

Please tell me whether any of these were problems for you at the LAST VISIT, and if so, whether they were a "MAJOR Problem" or a "MINOR Problem" for	Survey options:<br>0 - no problem<br>1- minor problem<br>2 - major problem
Availability:<br>Time you waited to see a provider<br>The hours of service at this facility, i.e., when they open and close	
Privacy:<br>Ability to discuss problems or concerns<br>Privacy from having others see the examination<br>Privacy from having others hear your consultation discussion	Scoring for analysis:<br>0 - no problem<br>1 - minor or major problem
Medicine availability:<br>Availability of medicines at this facility	
Cleanliness:<br>The cleanliness of the facility	
