# Supplementary material for: Factors associated with men’s health facility attendance as clients and caregivers in Malawi: a community-representative survey
Source: BMC Public Health. 2022 Oct 12;22:1904. doi: 10.1186/s12889-022-14300-8 (PMC9558411; doi:10.1186/s12889-022-14300-8)
Supplement: Supplementary file 3 — Supplementary Material 3 [file 12889_2022_14300_MOESM3_ESM.pdf]

Appendix: Predictive factors of any visit (client or guardian)

Variable			Any visit - univariate analyses			Likelihood of ANY visit - multivariate analysis	
	Overall		Attended any visit in last 12 months	No visit in last 12 months	P value	Odds ratio	95% CI
	n	(%)					
All men	1116	(100%)	919 (82%)	197 (18%)			
**Beliefs and Norms**							
Violence scale					0.00 ***	0.593 ***	0.415 - 0.848
Top 20%	272	(24%)	207 (76%)	65 (24%)			
Remaining 80%	844	(76%)	712 (84%)	132 (16%)			
Dominance scale					0.01 **	0.672 **	0.462 - 0.977
Top 20%	231	(21%)	177 (77%)	54 (23%)			
Remaining 80%	885	(79%)	742 (84%)	143 (16%)			
Women's roles scale					0.81		
Top 20%	315	(28%)	258 (82%)	57 (18%)			
Remaining 80%	801	(72%)	661 (83%)	140 (17%)			
Decision-making scale					0.07 *	1.326	0.887 - 1.983
Top 20%	308	(28%)	264 (86%)	44 (14%)			
Remaining 80%	808	(72%)	655 (81%)	153 (19%)			
**Sociodemographic Indicators**							
Age ‡					0.01 ***		
15-29 years	345	(31%)	277 (80%)	68 (20%)		0.907	0.480 - 1.713
30-49 years	393	(35%)	343 (87%)	50 (13%)		*reference*	
50+ years	378	(34%)	299 (79%)	79 (21%)		0.553	0.369 - 0.829
Household composition ‡							
Married					0.09 *	1.07	0.447 - 2.561
Married (monogamous or polygamous)	824	(74%)	688 (83%)	136 (17%)			
Not married	292	(26%)	231 (79%)	61 (21%)			
Children					0.06 *	1.494	0.583 - 3.827
Has children at home	820	(73%)	686 (84%)	134 (16%)			
No children at home	296	(27%)	233 (79%)	63 (21%)			
Distance from facility							
Dinstance from nearest public facility (km)	5.11		4.94	5.92	0.00 ***	0.963	0.914 - 1.015
Economic Indicators							
Assets (index created by PCA) - mean score	1.88		1.902	1.784	0.36		
Employment					0.26		
Formal or self-employment	663	(59%)	553 (83%)	110 (17%)			
Unemployed or ganyu (piece work)	453	(41%)	366 (81%)	87 (19%)			
Mobility					0.23		
More than 3 nights away in 6 months	299	(27%)	263 (88%)	46 (15%)			
Less than 3 nights away in 6 months	817	(73%)	666 (82%)	151 (18%)			
Savings					0.19		
Has savings	356	(32%)	301 (85%)	55 (15%)			
Does not have savings	760	(68%)	618 (81%)	142 (19%)			
Secondary school					0.05 **	1.323	0.848 - 2.064
Attended secondary school	228	(20%)	198 (87%)	30 (13%)			
Did not attend secondary school	888	(80%)	721 (81%)	167 (19%)			
Health status (self-described) ‡					0.2	0.699	0.434 - 1.124
Good or very good health	941	(84%)	769 (82%)	172 (18%)			
Poor or very poor health	175	(16%)	150 (86%)	25 (14%)			
**Health system Factors**							
Problems with quality (composite) - maximum 7	1.22		1.21	1.26	0.00 ***	0.322 **	0.126 - 0.824

* Significant at 0.10 (included in multivariate model)
** Significant at 0.05
*** Significant at 0.01
‡ Included in multivariate model as a control regardless of significance in univariate analysis
